# Combining causal and correlative approaches to discover biomarkers of response to paclitaxel

**DOI:** 10.18632/oncotarget.28549

**Published:** 2024-02-08

**Authors:** Alberto Moscona-Nissan, Karl J. Habashy, Victor A. Arrieta, Adam M. Sonabend, Crismita Dmello

**Affiliations:** ^1^Universidad Panamericana School of Medicine, Mexico City, Mexico; ^2^Department of Neurological Surgery, Feinberg School of Medicine, Northwestern University, Chicago IL 60611, USA; ^3^Northwestern Medicine, Malnati Brain Tumor Institute of the Lurie Comprehensive Cancer Center, Feinberg School of Medicine, Northwestern University, Chicago IL 60611, USA; ^4^PECEM, Facultad de Medicina, Universidad Nacional Autónoma de México, Mexico City, Mexico

**Keywords:** glioblastoma, predictive biomarker, CRISPR screen, paclitaxel

## Abstract

We recently discovered a putative paclitaxel response predictive biomarker for glioblastoma and breast cancer using the whole genome CRISPR knockout screen. The biomarker candidate was validated in two independent breast cancer patient cohorts that received taxane treatment. To further evaluate the potential application of this biomarker in the clinic for patients with glioblastoma, a prospective validation in cohorts of patients with glioblastoma is essential and will be performed as part of our ongoing phase II clinical trial (NCT04528680). The validation of novel biomarkers of susceptibility to therapy is critical to elucidate the efficacy signal of therapeutic agents. This is especially important in the context of glioblastoma, where therapeutic benefit is variable and unpredictable, leading to negative trials, yet the outcome of subset of patients has outperformed expectations.

## INTRODUCTION

### Repurposing paclitaxel drug for glioblastoma treatment

Paclitaxel (PTX) is a widely used and highly potent chemotherapeutic agent, being the basis of regimens to treat breast, pancreatic, ovarian, and lung non-small cell lung carcinomas [1]. However, it is estimated that half of the patients treated with PTX do not receive a therapeutic benefit, yet are exposed to its toxic effects, causing physical, psychological, and social discomfort [2, 3].

In glioblastoma (GBM) treatment, PTX has proven a high efficacy at nanomolar concentrations in achieving *in vitro* tumor cell death, with an IC50 1,400-fold lower than temozolomide, standard-of-care treatment [2–4]. However, minimal response to PTX in patients was demonstrated in several clinical trials [5–8]. PTX efficacy in patients is mainly limited due to factors such as inadequate blood-brain barrier (BBB) penetration and tumor heterogeneity [3].

### Barriers to paclitaxel treatment for glioblastoma and ways to overcome them

GBM treatment has faced important challenges due to several mechanisms of resistance that encompass intrinsic factors such as tumor isolation by the BBB and location within the brain; as well as tumor heterogeneity and immunosuppressive microenvironment [9]. Promising ways to overcome GBM resistance due to BBB isolation are being developed, these include convection-enhanced delivery, biodegradable wafers, peptide-drug conjugates, and low-intensity pulsed ultrasound administered with microbubbles (LIPU/MB) [10–13]. The latter strategy has been demonstrated safe in several clinical trials [11, 14]; and more recently, results from a phase I clinical trial where patients with recurrent GBM received paclitaxel or carboplatin after transient opening of the BBB with LIPU/MB illustrated that the strategy indeed results in a multifold increase in the drug concentrations in the brain [10].

Despite the extensive efforts to overcome the BBB and improve drug delivery, response to treatment in GBM remains unpredictable and importantly inconsistent across patients, highlighting tumor heterogeneity. In that context, identifying biomarkers predictive of response is crucial to refine patient selection, improve treatment efficacy, and prolong survival in patient subpopulations. Biomarker discovery is leading to a paradigm shift in cancer treatment by providing powerful information to improve diagnostic accuracy, predict treatment response and determine prognosis [15].

### Discovery of predictive biomarkers through CRISPR screen and combination of correlative evidence from breast cancer

**Figure 1:**
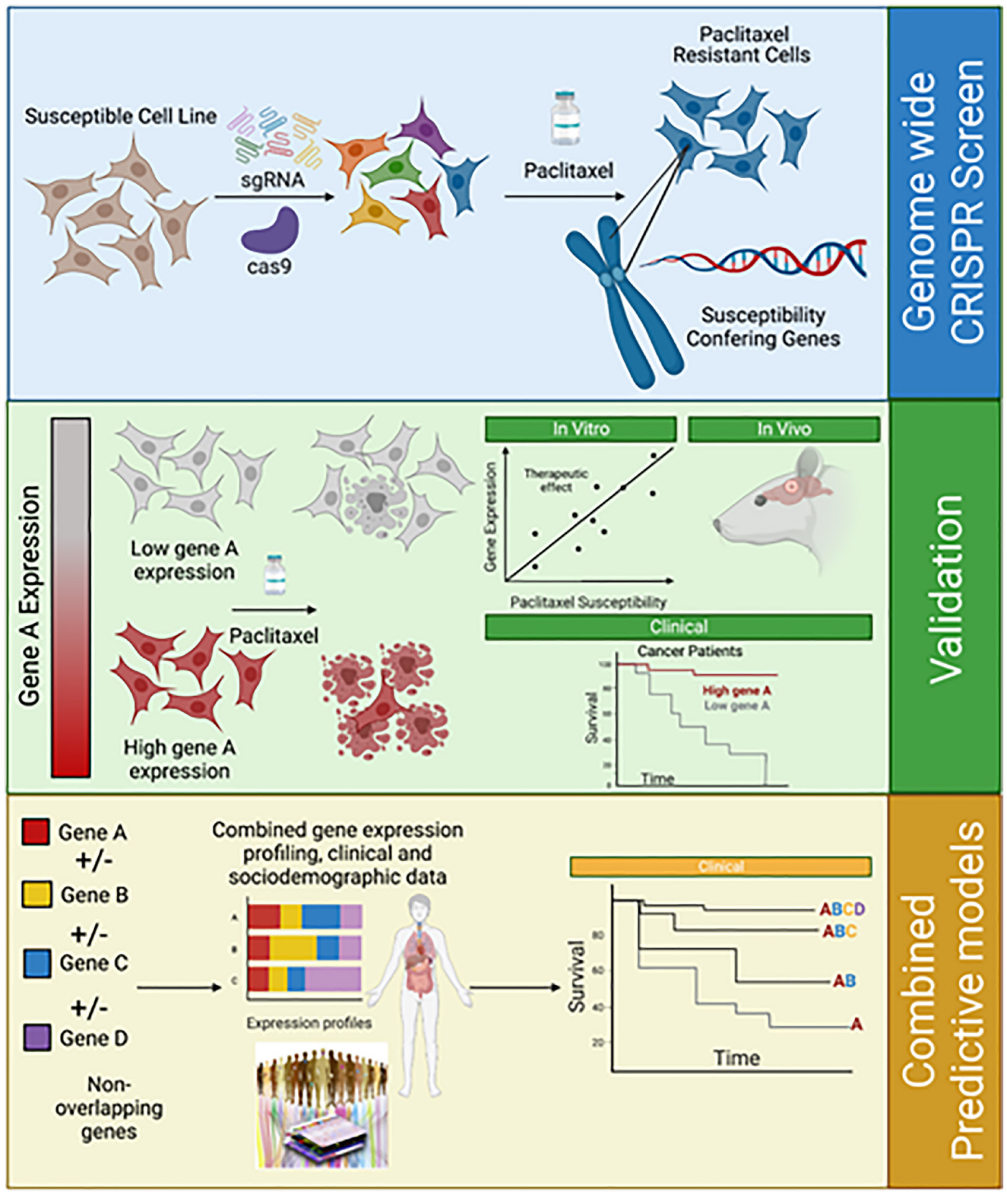
From genome-wide CRISPR screen to the creation of combined predicting models. A whole-genome CRISPR/Cas9 knockout was performed using single-guide RNA in selected cells. Genomic DNA was extracted in some cells before a treatment course with paclitaxel in dimethyl sulfoxide or dimethyl sulfoxide alone for 21 days. Cells were harvested on days 0, 14, and 21. Genomic DNA was extracted and amplified. After paclitaxel treatment, expansion of resistant clones and susceptibility-conferring genes were identified. In order to validate the role of a specific gene after its identification, differential gene expression in clones was correlated with paclitaxel susceptibility *in vitro* and *in vivo* assays (by seeding heterotopic tumors in mice, which were further treated with albumin-bound paclitaxel). In order to validate the reproducibility and generalizability of a biomarker, validation in independent cohorts should be performed. Furthermore, studying the combination of non-overlapping biomarkers’ expression, in addition to clinical and sociodemographic data could generate predictive models for paclitaxel susceptibility.

Through techniques such as CRISPR genetic knockout (KO) and RNA interference screens in cancer cells, it has been possible to target genes whose depletion or absence modifies susceptibility patterns to chemotherapeutic agents [16, 17]. We identified 51 genes that influence PTX susceptibility in gliomas through an unbiased CRISPR KO screen in H4 and GBM6 cell lines, which were treated with PTX or DMSO for 21 days (Figure 1). Considering basal gene expression and through guide RNA comparison between PTX-treated versus DMSO-treated samples using differential gene expression analysis, 51 putative genes involved in PTX susceptibility were identified. The above-mentioned have an implication in pathways like NFkB, toll-like receptor, and MAPK signaling, transcriptional misregulation, and apoptosis [2].

A Cox analysis was performed using two independent breast cancer datasets, The Cancer Genome Atlas (TCGA) and Gene Expression Omnibus (GEO). Breast cancer was studied given PTX routine treatment in this neoplasm, robust studies of patient outcomes, and possible immediate application after the discovery of a predictive biomarker. Through TCGA, gene expression and overall survival (OS) among patients with breast cancer treated with taxanes, other chemotherapeutic agents, and untreated patients were assessed. Five of the 51 previously identified genes displayed a significant interaction between gene expression and OS in taxane-treated patients. Among them, the signal sequence receptor 3 (SSR3) gene showed to be predictive of PTX susceptibility in TCGA, giving patients a favorable OS and higher relapse-free survival when treated with taxanes [2]. In the GEO dataset, which is composed of patients who did not receive hormonal therapy or chemotherapy, SSR3 did not show to be predictive of relapse-free survival [2].

Heterotopic tumors were seeded in mice through intracranial and mammary fat pad injections and further treated with PTX or phosphate buffer solution (Figure 1). Cell lines’ susceptibility to PTX was measured through determination of the area under the curve values and SSR3 expression was studied by Western blot analysis [2]. A negative correlation was found between higher SSR3 expression and PTX resistance in GBM and breast cancer cell lines. SSR3 KO cells showed a decreased susceptibility to PTX, while cells overexpressing SSR3, had increased susceptibility to PTX [2]. In order to confirm the role of SSR3 in PTX susceptibility, single-gene KO for SS3 and overexpression induction through a plasmid were performed. SSR3 KO in PTX-sensitive breast cancer (MDA-MB-468) and glioma H4 cells resulted in resistance to PTX. Correspondingly, SSR3 overexpression rendered PTX-resistant cells (GBM6) susceptible to PTX [2].

### The role of SSR3 gene in paclitaxel susceptibility and implied biological pathways

SSR3 gene codifies the gamma subunit of the signal sequence receptor (SSR) complex, a glycosylated membrane receptor located at the endoplasmic reticulum (ER), consisting of four different subunits associated with protein translocation across the membrane of the ER [18]. SSR complex is also known as TRAP complex, essentially involved in folding and transport of protein to the ER. Its functions are related to the unfolded protein response (UPR) pathway, which reduces the amount of unfolded proteins in the cell under stressful conditions. IRE1, PERK, and ATF6 are signaling pathways implicated in UPR activation in order to achieve protein homeostasis [19]. IRE1 (Inositol/requiring enzyme type 1) is a serine/threonine kinase that has been found in animals, plants, and yeast. IRE1 activity increase has been confirmed in neoplastic, inflammatory, metabolic, and neurodegenerative disorders [20].

We discovered a positive correlation between SSR3 expression and IRE1a levels in glioma PDX cells. A deep interaction between SSR3 and IRE1a can be explained by ER stress response and transport machinery. SSR3 KO in H4 cells led to a decrease in phosphorylation levels in IRE1a in the presence of PTX treatment [2]. Moreover, KO of IRE1a in PTX-sensitive H4 cells provided them resistance to PTX. In the GBM6 cell line (characterized by basal resistance to PTX), induction of SSR3 over-expression conferred cells susceptibility to PTX. However, inducting IRE1a KO rendered cells resistant to PTX. Conversely, IRE1a KO in PTX-resistant GBM6 cells (SSR3 low), conferred an increase in PTX resistance [2].

### Paclitaxel susceptibility biomarkers involved in microtubule function

Given that PTX’s primary mechanism of action relies on microtubule stabilization, genes coding for proteins involved in microtubule assembly and cytoskeleton biology have been investigated as potential biomarkers of response to PTX. Rodrigues-Ferreira [21] investigated the predictive value in breast cancer of 280 genes encoding proteins involved in microtubule functions; finding the MTUS1 gene and its codified protein ATIP3 as predictive biomarkers to taxanes [21]. Low ATIP3 levels correlated with a better response to neoadjuvant chemotherapy, by inducing proapoptotic effects, mitotic abnormalities, and aneuploidy [21].

Additionally, since 2005, Rouzier [22] demonstrated the predictive potential of microtubule-associated protein tau (MAPT). Differential gene expression was correlated with a pathological complete response (pCR) to PTX treatment. Tau protein was negative in 74% of pCR cases, predicting a higher pCR rate [22]. Down-regulation of tau through siRNA increased PTX sensitivity in breast cancer. One of the mechanisms proposed is that in presence of high tau protein expression, microtubules are physiologically stabilized, reducing PTX binding to tubulin [22]. Regarding microtubule assembly and SSR3 gene, we found no correlation between these mechanisms [2].

### Validation and consideration of multiple genes and models to advance the precision of prediction

Beyond SSR3, the expression of other genes such as CEP63, IRAK4, TMEM131, MBNL1, ZBTB20, and TDRD1 demonstrated a significant correlation with OS in taxane-treated patients [2]. The predictive potential of this set of genes remains to be validated. While other potential biomarkers of response to PTX have been explored, their use in the clinic is still limited due to the less informative results in external validation cohorts. In fact, after identifying a potential predictive biomarker in a training cohort of patients, it is crucial to validate the reproducibility and generalizability of the finding in an independent cohort (Figure 1). Currently, the correlation between patients’ OS and SSR3 expression is under study in the Ultrasound-based Blood-brain Barrier Opening and Albumin-bound Paclitaxel and Carboplatin for Recurrent Glioblastoma (SC9/ABX) Phase 2 trial at Northwestern University (NCT 04528680).

Validation studies in independent sample cohorts such as clinical trials or retrospective analysis employing statistical and bioinformatic evaluation should be performed through robust assays, reviewing technical reproducibility, patient-to-patient variations, and other possible sources of bias [23, 24].

As shown in Figure 1, further directions encompass generating predictive models that build on individually validated biomarkers to integrate them into a more comprehensive assay. As such, the expression of several non-overlapping histologic and molecular biomarkers can form “gene signatures” that can be combined with radiographic and physiological biomarkers as well as patient demographics to predict patients’ clinical outcomes with higher reliability [24, 25].

### Predictive biomarker discovery leading to a paradigm shift in cancer treatment

The discovery of predictive biomarkers to PTX vulnerability as SSR3 promises to significantly impact cancer treatment. In fact, breast, lung, ovarian, and pancreatic cancer, which are among the leading causes of death worldwide and represent a huge burden to healthcare systems, rely heavily on treatment with PTX. Validation of biomarkers of response to this therapy is therefore crucial to refine patient selection for PTX therapy and provide personalized treatment based on the predicted response. As equally important as treating potentially responsive patients, avoiding treatment in potentially resistant patients reduces unnecessary toxicity, decreases the time to trial of another therapy, potentially improves health outcomes and quality of life, and significantly alleviates the healthcare burden [26].

Precision and personalized medicine can lead to a transition from a stochastic treatment response into predictable scenarios. Further identification of predictive biomarkers, validation, and study of combinations as predictive models is critical to generate a greater impact that can be translated to the bedside of patients.
